# Case report: Tall cell carcinoma with reversed polarity of the breast: an additional case and review of the literature

**DOI:** 10.3389/fonc.2024.1302196

**Published:** 2024-02-16

**Authors:** Zi Lei, Ying-Xia Wang, Zhi-Yuan Wang, Cheng-gang Yang, Guo-Qing Pan

**Affiliations:** ^1^Department of Pathology, First Affiliated Hospital of Kunming Medical University, Kunming, China; ^2^Department of Pathology, Third Affiliated Hospital of Kunming Medical University, Kunming, China

**Keywords:** breast cancer, Tall cell carcinoma with reversed polarity of the breast, clinicopathological features, histological characteristics, differential diagnosis

## Abstract

**Objective:**

The aim of this report was to comprehensively investigate the clinicopathological features, histological characteristics, and differential diagnosis of tall cell carcinoma with reversed polarity of the breast (TCCRP) to enhance the understanding of this tumour for precise therapeutic interventions.

**Methods:**

The clinicopathological characteristics and differential diagnosis of a patient with TCCRP were retrospectively analysed, and a systematic literature review was extracted from relevant published studies on PubMed.

**Results:**

All patients included in the study were female, with a median age of 51 years. Microscopically, the tumour cells exhibited a solid papillary growth pattern with tall columnar morphology and reversed nuclear polarity. Immunohistochemistry revealed that the tumours were triple-negative breast cancer (negative for ER, PR, and HER-2), with a low Ki-67 proliferation index. Different degrees of expression were observed for CK7, Calretinin, and S-100 markers; however, CK5/6 showed high expression levels.

**Conclusions:**

TCCRP is an uncommon invasive carcinoma subtype found in the breast. Its histological morphology resembles that of tall cell subtype papillary thyroid carcinoma. Accurate diagnosis requires the integration of histomorphological assessment along with immunohistochemistry and molecular genetics analysis.

## Introduction

TCCRP is a rare type of invasive breast carcinoma. In 2003, Eusebi et al. reported five cases of breast tumours with histological features resembling those of tall cell variant papillary thyroid carcinoma (1). All patients were female, with an average age of 63 years, and no history of thyroid disease. The tumour cells exhibited solid or papillary growth patterns, with some areas showing a follicular structure reminiscent of the thyroid and containing eosinophilic glial material. These tumour cells displayed a columnar or cuboidal morphology, characterized by an eosinophilic cytoplasm due to abundant mitochondria. Most nuclei were oval-shaped with nuclear grooves observed in many instances. Some tumours exhibited intranuclear pseudoinclusions, psammoma bodies, and granular calcification. Immunohistochemical analysis revealed positive staining for CK7 and mitochondrial antibodies but negative staining for TG, TTF-1, oestrogen receptor (ER), and progesterone receptor (PR). Subsequent studies have contributed to our understanding of this tumour’s morphology, immunophenotype, molecular genetic alterations, and biological behaviour (2–15). Notably, Chang et al. identified nuclear polarity inversion as a distinctive feature of these tumour cells (4). Chiang et al., through high-throughput whole-exome sequencing and targeted sequencing techniques, discovered an IDH2 R172 hotspot mutation in these tumors (8). Consequently, the name solid papillary carcinoma with reverse polarity (SPCRP) was coined, and the WHO (2019) designated this entity tall cell carcinoma with reversed polarity (TCCRP).

TCCRP is a rare disease that has been reported in only a limited number of studies, thus, it remains poorly understood by clinicians and pathologists. This study aimed to enhance the comprehension of this tumour and improve the accuracy of pathological diagnosis through an analysis of its clinicopathological features and a comprehensive literature review.

## Materials and methods

### Cases

One TCCRP case was obtained from the Pathology Consultation Center of Yunnan Province, and it met the diagnostic criteria for TCCRP as outlined in the WHO classification of breast tumours (2019).

A PubMed (http://www.ncbi.nlm.nih.gov/pubmed/) literature search was conducted using a combination of various keywords related to the title/abstract, such as “Breast tumor resembling the tall cell variant of papillary thyroid carcinoma,” “Tall cell variant of papillary breast carcinoma,” “Breast cancer with altered nuclear polarity,” and “Solid papillary carcinoma with reverse polarity of the breast.” Relevant published studies were reviewed, and necessary clinicopathological data were extracted. A total of 81 cases retrieved from the literature were included in our review.

### Intraoperative pathological examination of frozen sections

Samples were obtained by surgical excision of breast tissue; immediately a thorough examination for the presence of nodules was performed. The nodule lesion was cut into a tissue block measuring approximately 1.5cm × 1.5cm × 0.2 cm, embedded it in a freezing agent, and placed in a -20°C frozen slicer. After embedding, the tissue was sliced into 5 mm sections, fixed them in alcohol ether for one minute, and subsequently stained with haematoxylin eosin (HE).

### Paraffin pathological section examination

After sectioning the tissue, the remaining tissues were fixed in 10% neutral formaldehyde for 6-8 hours and dehydrated overnight. Subsequently, the wax blocks were cut into thin sections measuring 4 millimetres using a wax embedding machine. These sections underwent a baking process lasting for 30 minutes, followed by dewaxing and removal of benzenes through washing. Haematoxylin and eosin stains were applied prior to another round of washing, dehydration, transparentization, and sealing of the samples. Finally, morphological characteristics were observed under a light microscope.

### Immunohistochemical staining

Immunohistochemical detection was performed using the optimized EnVision two-step method and DAB staining. The primary antibodies utilized for IHC were obtained from Fuzhou Maixin Biotechnology Development Co., LTD, except IDH2 R172S. The immunohistochemical analysis of IDH2 R172 was performed using a monoclonal antibody raised against IDH2 R172S (clone 11C8B1; NewEast Biosciences, Malvern, PA) (**Table 1**).

**Table 1:** Clones, and Dilution of primary antibodies for IHC.

Aigen	Clone	Dilution
Mammaglobin	MAB-0561	1:400
TTF-1	MAB-0677	1:400
p63	MAB-0694	1:400
TG	MAB0797	1:400
SMMHC	MAB0121	1:400
E-Cadherin	MAB0738	1:400
P120	MAB1077	1:400
ER	kit-0012	1:400
PR	kit-0013	1:400
AR	RMA-0807	1:400
HER-2	kit-0043	1:400
Ki-67	Ki-67	1:400
Syn	MAB-0742	1:400
CgA	MAB0707	1:400
CD56	MAB0743	1:400
CK5/6	MAB-0744	1:400
Calretinin	MX027	1:400
IDH2 R132	clone 11C8B1	1:2000

## Results

### Clinical presentation

The present case involved a 65-year-old woman who presented with a persistent left breast mass for over four months. Four months prior, the patient incidentally discovered a thumb-sized mass in her left breast accompanied by occasional tingling and no bloody nipple discharge. Physical examination revealed well-developed and symmetrical breasts without dimpling or skin changes resembling an orange peel. No bleeding or discharge was observed upon bilateral nipple compression. A large, hard-textured mass measuring less than 2 cm×2 cm was palpable in the inner upper quadrant of the left breast, displaying unclear boundaries and limited mobility. Mammography indicated an internal space-occupying lesion in the left breast suggestive of malignancy and possible breast cancer (BI-RADS: 4C). B-ultrasound demonstrated a solid mass lesion of unknown nature in the left breast (BI-RADS: 4C), raising suspicion of breast cancer. Contrast-enhanced ultrasound showed no substantial lesions with an unknown nature in the left breast (BI-RADS: 5), further supporting consideration of breast cancer. Magnetic resonance imaging (MRI) confirmed a malignant-appearing medial mass on the left side highly indicative of breast cancer (BI-RADS: 5). The patient requested surgical removal of the mass directly, so a rapid intraoperative procedure was performed. Detailed clinical data from this case as well as previously reported cases are summarized in **Table 2**.

**Table 2:** Summary of clinical data in the TCCRP literature.

Literature	Cases	Age (The average)	Paimary site(cases)	Tumor size(cm)	Lymph node metastasis	Insitu/infiltrated	Treatment	Follow-up (average month)
Present study	1	65	L	2.0	NA	CIS	Simple excision	48, NED
Eusebi et al.2003 (1)	5	56-74(63)	L (2) R (2) NA(1)	0.8~2.0	NA	CIS (1) MIC (2) IC (2)	Expanded resection	26-108(54)
Cameselle-Teijeiro et al 2006 (2).	1	64	R	4. 1	Yes (1)	IC	Single pure resection+C/XRT /HT	32 bone metastases
Tosi et al.2007 (3)	4	45-80	R(4)	2-5	Yes (1) No (3)	CIS (2) IC (1) NA (1)	Quadrantectomy (4)	5-120 (35. 8) NED (4)
Chang et al.2007 (4)	1	66	L	1.1	No	IC	Segmental resection	12, NED
Masood et al. 2012 (5)	1	57	L	3	No	IC	Simple excision	NA
Colella et al. 2015 (6)	1	79	R	8.5	No	MIC	Quadrantectomy	18, NED
Baohua Yu et al 2015 (7).	1	53	R	1.8	NA	IC	Lumpectomy	NA
Chiang, et al.2016 (8)	13	51-79 (65)	L (8) R (5)	0. 6-1. 8	No (8) NA (5)	IC (13)	S/XRT/C (1) S/XRT (2) S/C (1) S (1) NA (8)	12-77(33. 7) NED (7) NA (6)
Foschini* et al.2017 (9)	13	48-85 (63)	L (7) R (6)	0. 6- 2. 5	Yes (1) No (4) NA (8)	IC (13)	Mastectomy (12) Mastectomy + C /XRT (1)	24-132 (77), recurrence at 60 months with axillary lymph node metastasis (1) NED (10) NA (2)
Bhargava et al. 2016 (10)	3	48-77 (63)	L (2) R (1)	0. 9 -1. 7	NA (3)	IC (3)	NA (3)	19 NED (2) NA (1)
Pitino A et al.2017 (16)	1	65	R	0.7	No	IC	Quadrant resection	34 NED
Gai,L.et al.2018 (17)	1	55	R	NA	No	IC	Simple excision	NA
Alsodoun,N.et al.2018 (11)	9	52-75 (66)	L (5) R (4)	0.9-4	No (9)	IC (9)	segmental resection (9)	53 NED (1) NA (8)
Lozada,J.R.et al.2018 (12)*	6	58-85 (65)	NA (6)	0.6-2.1	No (2) NA (4)	IC (6)	NA (6)	NA(6)
Zhong,E.et al.2019 (13)^※^	9	63-79 (70)	L (5) R (4)	0.7-1. 8	NA (9) NA (9)	IC (9)	NA (9)	16-64 (32) NED (unknown)
Ding,L.M.et al.2019 (14)	1	70	L	1. 6	NA (1)	IC	Simple excision	9, NED
Pareja,F. et al.2020 (15)^#^	14	46-85(63)	NA (14)	0. 6-2. 6	NA (6) NA (8)	IC (14)	Partial resection (2) partial resection +XRT(3) mastectomy (1) NA(8)	NA (14)
Haefliger, S. et al.2020 (18)	1	60	R	0.8	No	IC	Tumorectomy and sentinel lymph node excision	8, NED
Trihia HJ et al.2021 (19)	1	71	R	0.9	No	IC	Partial resection	18, NED
Zhang, X. et al. 2021 (20)	1	45	L	1.2	No	IC	Local excision sentinel and lymph node biopsy	NA
Cui LJ, et al. 2021 (21)	1	62	L	2.0	NA	IC	Mastectomy	6, NED
Jassim, M. et al.2021 (22)	1	40	R	5.5	No	IC	modified radical mastectomy with axillary clearance	6, NED

NA, no access to information; L, left; R, right; CIS, carcinoma in situ; IC, invasive carcinoma; MIC, minimally invasive carcinoma; NED, disease-free survival; C, chemotherapy; XRT, radiation therapy; HT, endocrine therapy; S, surgery. *Two cases in the Foschini group overlapped with those in the Eusebi and Tosi groups. ^※^One case in the Zhong group was duplicated with that in the Chiang group. ^#^ Five cases from Pareja's study were replicated in either Lozada's, Bhargava's, or Zhong's studies.

### Intraoperative frozen section microscopic features

**Figure 1:**
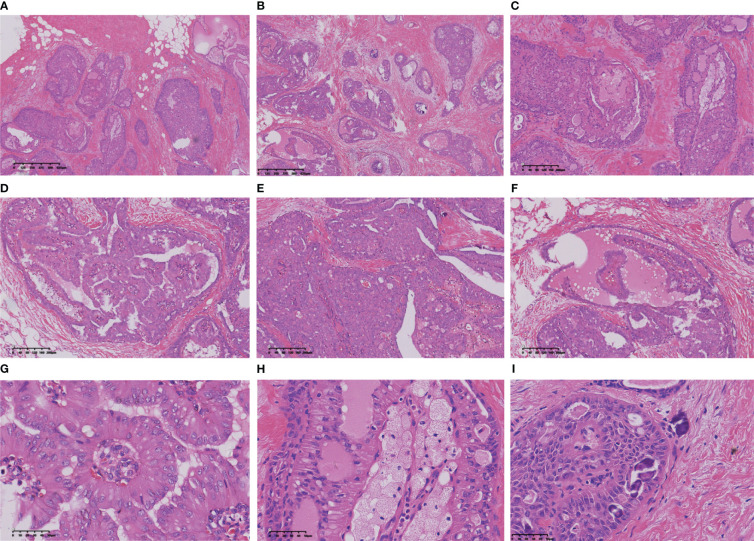
The nests of tumour cells showed expansion infiltrating growth **(A, B)**, interstitial collagenization **(C)**, intraductal papillomatous structures **(D)**, and solid papillomatous structures **(E)**. The tumour cells form a glandular cavity with glial secretions **(F)**. The tumour cells on the papillary surface are columnar, with the nucleus adjacent to the margin of the cavity and away from the basement membrane **(G)**. More foam cells were aggregated in the papillary axis **(H)**. Sand particles found in tumour nests and stroma **(I)**.

The intraoperative frozen sections revealed tumour cell nests exhibiting swelling and interstitial fibrosis (**Figures 1A, B**). In certain regions of the tumour, structures resembling intraductal papilloma were observed, accompanied by the accumulation of foam cells along the axis of the papilla (**Figures 1C–E**), making it challenging to differentiate them from intraductal papilloma using frozen section analysis. Some of these formations exhibited a sieve-like structure, with gum-like secretions visible within the cavity and sand bodies present in focal areas (**Figures 1F–I**). Notably, the columnar tumour cells appeared distant from the basement membrane (**Figure 1G**).

### Postoperative paraffin section microscopic features

**Figure 2:**
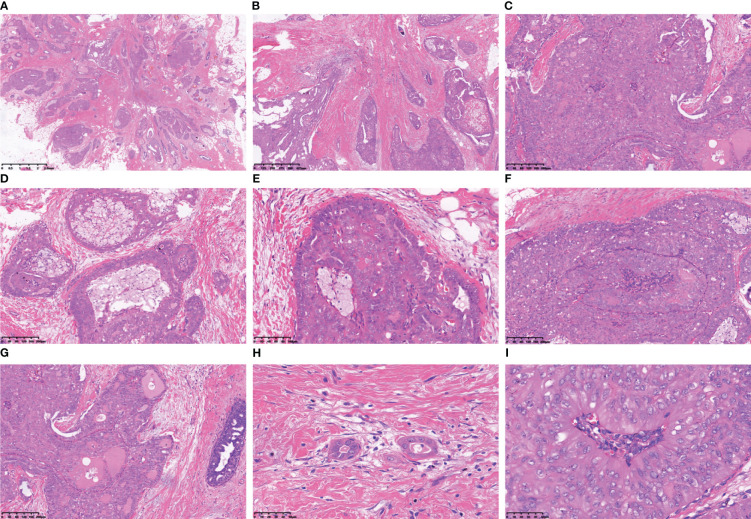
The tumour cells arrange themselves in nests and demonstrate infiltrative growth that expands extensively, accompanied by stromal collagenization **(A, B)**. The tumour cells exhibited a cohesive papillary architecture **(C)**, with an increased accumulation of foam cells within the central axis of the papillae **(D)**. The cells surrounding the tumour nest frequently exhibit a columnar morphology, being arranged in a perpendicular palisade orientation relative to the basement membrane **(E)**. The tumour cells form intraductal papillomatous structures characterized by intricate and heterogeneous papillary branching **(F)**. Tumour cells form secondary glandular cavities/ethmoids of varying sizes, and the quantity of glial secretions within these cavities varies **(G)**. Tubular-like and single follicular tumour clusters were observed within the fibrous interstitium **(H)**. The neoplastic cells lining the peri-lumen or papillary surface exhibited a columnar morphology, with their nuclei positioned adjacent to the lumen but distant from the basement membrane **(I)**.

Macroscopically, the tumour presents as a solid nodular mass with grey-white areas, exhibiting a hard to firm consistency and indistinct margins from surrounding tissue. All reported tumors exhibited identical histological characteristics, with tumour cells forming round or ovoid nests of varying sizes and displaying expansive infiltrative growth (**Figure 2A**). Some of the tumour clusters exhibited irregular nests and twisted branches or structures, while the tumour stroma displayed hypocellular collagenized fibres or mild desmoplastic reactions (**Figure 2B**). The nests of tumour cells demonstrated three primary structures, including a solid papillary structure, an intraductal papilloma-like structure, and a papillocribriform structure. Specifically, the solid papillary structure revealed that the tumour cells proliferated around a fibrovascular axis with varying thickness (**Figure 2C**). Oedema, hyalinization, capillary congestion, and lymphocyte infiltration were observed in the stroma of the fibrovascular axis, while foam cells accumulated in the papillary axis (**Figure 2D**). In solid tumours, the cells surrounding the tumor nests and axis often exhibited a columnar morphology and were arranged in a palisade pattern perpendicular to the basement membrane (**Figure 2E**). The presence of an intraductal papilloma-like structure indicated the formation of a guideway-like arrangement around the tumour nests, with its inner wall lined with a single layer of columnar cells. Within the lumen, tall columnar and cuboid tumour cells covered the surface of the fibrovascular axis, forming intricate branching papillae (**Figure 2F**). Additionally, localized areas may exhibit hobnail changes. The proposed papillocribriform structure suggests fusion of neoplastic papillae, resulting in the formation of tension round/oval or irregular staghorn-shaped secondary gland cavities/sieves by tumour cells. Variable amounts of pink secretions were observed within the glandular cavities/ethmoid holes, some of which exhibited absorption vacuoles surrounding the secretions, resembling changes seen in thyroid follicles (**Figure 2G**). Some glandular cavities/ethmoids lacked secretion or showed calcifications. Additionally, focal nests of tubular and monofollicular tumour clusters were identified, predominantly composed of columnar (tall columnar, short columnar, or obese columnar) and polygonal tumour cells, characterized by abundant or moderate cytoplasm that appeared eosinophilic, reddish, or clear (**Figure 2H**). The nuclei of these tumour cells displayed a round, oval or irregular short rod-shaped morphology with finely granular chromatin and small- to medium-sized nucleoli that appeared reddish in colour. Mitotic figures were rare and conspicuous nuclear grooves were present; scattered intranuclear pseudoinclusion bodies were also observed. Reversal of nuclear polarity was noted in (tall) columnar cells lining the surface of glandular lumens or branching papillae where their nuclei resided close to the lumens but away from the basement membrane (**Figure 2I**).

### Immunophenotypic characteristics

**Figure 3:**
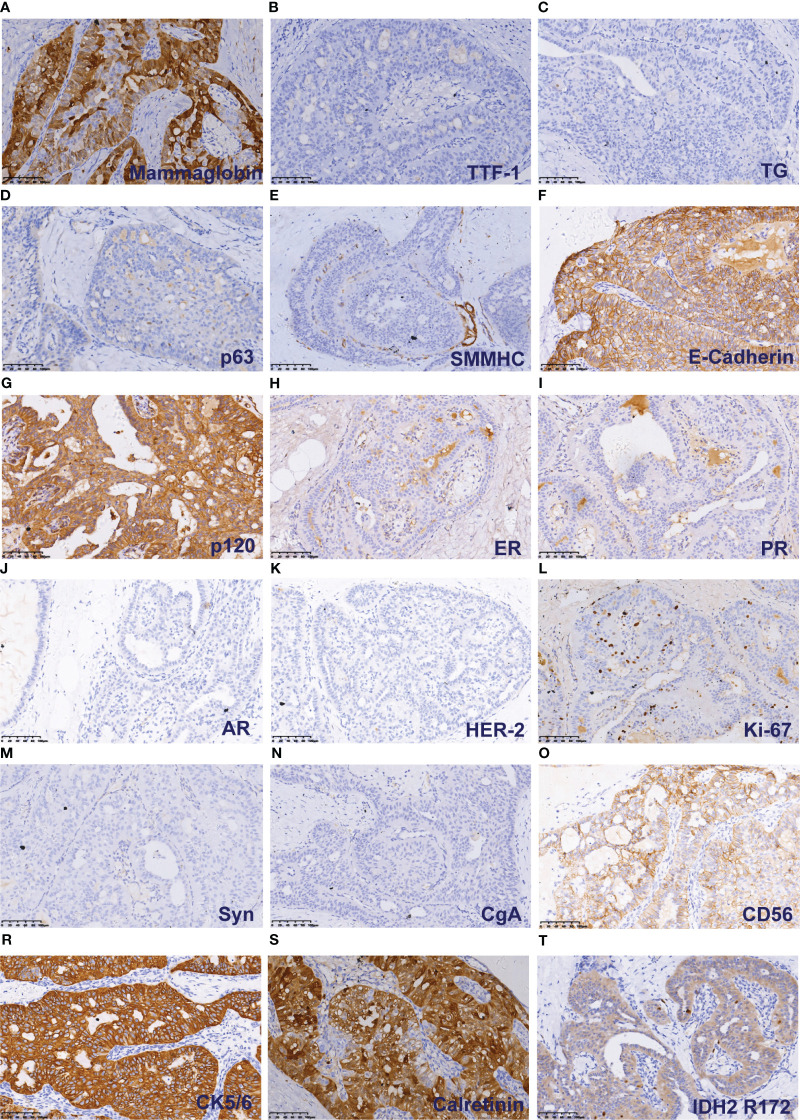
Immunohistochemical results of TCCRP. The cells were cytoplasmic positive for Mammaglobin **(A)**. The cells were negative for TTF-1 and TG **(B)**. The cells were negative for p63 and SMMHC **(D, E)**. The cells were membrane positive for E-Cadherin and p120 **(F, G)**. The cells were negative for ER, PR, AR and HER-2 **(H–K)**. The number of Ki-67 positive cells was <5% **(L)**. The cells were negative for Syn and CgA **(M, N)**. The cells were membrane positive for CD56, CK5/6, and Calretinin **(O-S)**. The cells were cytoplasmic positive for IDH2 R172 **(T)**.

Immunohistochemical staining of tumour cells revealed positive expression of Mammoglobin (**Figure 3A**), and the absence of TTF-1 and Thyroglobin (TG) immunoreactivity suggested that the primary origin of the tumor cells is in the breast (**Figures 3B, C**). P63 and SMMHC exhibited negative staining patterns indicative of myoepithelial cell disappearance surrounding the tumor (**Figures 3D, E**). E-Cadherin and p120 protein were positively expressed and localized in the cell membrane (**Figures 3F, G**). The lack of ER, PR, AR and HER-2 indicated the tumour was triple-negative breast cancer (**Figures 3H–K**). A Ki-67 level of approximately 3% implied a low proliferation index for the tumour cells (**Figure 3L**). Additionally, varying degrees of positivity were observed with CD56 along with CK5/6 and Calretinin (**Figures 3O-S**), but Syn and CgA were negative (**Figures 3M, N**). IDH2 R172 positivity indicated IDH2 172 mutation (**Figure 3T**).

## Discussion

TCCRP is a relatively rare subtype of breast cancer, with only 14 cases reported in the largest series (15). Due to its unique clinicopathological and molecular genetic characteristics, TCCRP has been classified as an independent subtype of “rare and salivary gland-type tumours” in the WHO (2019) classification of breast tumors to avoid confusion with breast papillary tumours and thyroid metastatic tumors (23).

A summary of the cases in this cohort and a literature review (**Table 2**) indicated that TCCRP is more prevalent among elderly females, with palpable breast masses as the primary clinical manifestation. TCCRP typically presents as small lesions (primarily T2), appearing as grey-white solid nodules with well-defined margins and a firm texture. The initial study conducted by Eusebi et al. strongly emphasized the histological similarities between TCCRP and papillary thyroid carcinoma, including the presence of papillary and follicular structures, columnar cells with nuclear grooves and intranuclear pseudoinclusion bodies, glial-like secretions, and calcification (1). In 2016, Chiang et al. identified a characteristic IDH2 R172 mutation in TCCRP and reported that the tumour exhibited distinct pathological features, including a solid papillary structure, reversal of nuclear polarity, accumulation of foam cells in the papillary axis, lack of myoepithelium and high expression of CK5/6 (8).

This group of studies demonstrated that TCCRP exhibits three main structural patterns, including a solid mastoid head structure, a papilloma-like structure, and a papillocribriform structure. All tumours contain cribriform structures with varying proportions. While the solid papillary structure is the primary structural pattern in TCCRP, some tumours also exhibit morphological changes similar to thyroid papillary carcinoma. Familiarity with the diverse morphological changes in TCCRP can help prevent missed diagnoses when limited material is available, such as that obtained with biopsy. Notably, atypical lesions may occur in TCCRP; for example, polygonal rather than columnar tumour cells may be present in some solid mastoid nests and nuclear polarity reversal may not always be significant or relatively limited (13), while complete lack of glial follicular changes and calcification are possible.

In the early literature, reported cases of TCCRP may solely represent *in situ* lesions; however, the 2019 Classification of Breast Tumours clearly categorizes TCCRP as a rare subtype of invasive breast adenocarcinoma. It is plausible that previously reported cases classified as *in situ* might have been misidentified. Nevertheless, if encountering hyperplastic breast lesions with an intact myoepithelial layer during routine practice, the likelihood of diagnosing TCCRP is diminished.

Several studies have shown that TCCRP stably expresses mitochondria (95%) due to the rich cytoplasm of mitochondria (1, 3, 9, 16). However, the infrequent use of this antibody in pathological laboratories poses a challenge to its accessibility. Recent studies conducted by Alsadoun et al. have demonstrated that TCCRP exhibits high expression of calretinin (clone number PAD: DC8), with only two cases of papilloma in the control group showing focal weak positive staining, indicating that calretinin is a useful marker for differential diagnosis (11). Pareja et al. reported that 10 tumours exhibited diffuse (5 cases) or focal (4 cases) positivity for calretinin (clone number SP65) (15). TCCRP demonstrated positive staining for GATA-3, GCDFP-15, MMT, and CK7 in this cohort; however, it was negative for TG, TTF-1, and PAX8 markers, providing support for its breast origin rather than originating from the thyroid gland.

The elevated expression levels of basal CK5/6 and CK34βE12 have diagnostic implications in TCCRP cases with histological grades 1 or 2. Analysis of tumour biomarkers demonstrated predominantly negative or low expression levels of hormone receptors and Her2 in TCCRP, where over half of the cases were categorized as triple-negative subtype. In published studies, the reported Ki67 proliferation index for TCCRP ranged from 1% to 31%, with a majority showing a positive index below 5%. Revised sentence: Early studies indicated that TCCRP lacks the typical molecular genetic alterations observed in papillary thyroid carcinoma, such as BRAF and RET gene mutations. Recently, Chiang et al. identified the IDH2 R172 mutation in TCCRP (10/13 cases) using high-throughput sequencing technology (8). Subsequent investigations and case reports by these researchers have confirmed that this gene mutation is a frequent occurrence in TCCRP (with R172S being the most common) (8, 10–15), which is not present in classic solid papillary carcinoma, intraductal papilloma with usual hyperplasia, encapsulated papillary carcinoma or invasive micropapillary carcinoma (11, 12, 15). Thus, this mutation holds significant diagnostic value.

TCCRP should be distinguished from other diseases, such as metastatic papillary thyroid carcinoma. A comparative study by Zhong et al. found that a solid structure, histiocyte aggregation, and nuclear polarity reversal were specific to the diagnosis of TCCRP (13).However, history, tissue origin, and IHC staining can aid in diagnosing metastatic carcinoma. Furthermore, reports of breast metastasis from classic or high cell subtype papillary thyroid carcinoma are limited to case studies (13). The intraductal papilloma with usual hyperplasia exhibited a papillary structure, accompanied by foam cell aggregation in the papillary axis. Additionally, it displayed features of usual hyperplasia, a solid growth pattern with nuclear groove formation, and intranuclear pseudoinclusion bodies. Immunohistochemical staining for CK5/6 showed positive results, while myoepithelial cells were identified to support the diagnosis of papilloma. The cells of classic solid papillary carcinoma exhibit polygonal or spindle-shaped morphology, accompanied by varying degrees of intracellular and extracellular mucus secretion. They demonstrate high expression levels of neuroendocrine markers and hormone receptors, while testing negative for CK5/6. The absence of nuclear polarity flip and glial follicle-like structures serve as distinguishing features from TCCRP. Secretory carcinoma, also observed in adults, may exhibit microcystic, solid, or even papillary structures. The content within the cystic cavity resembles thyroid follicular colloid secretion; however, the immunophenotype (e.g., triple negative or receptor low expression and CK5/6 positivity) is akin to TCCRP. Bhargava et al. employed FISH detection of the ETV-6 gene in one case of TCCRP and secretory carcinoma (yielding negative results) (10). A Solid mammary head, columnar cells, and reversal of nuclear polarity support the diagnosis of TRRCP while S-100 positivity is observed in TCCRP (10, 17). Cystic hypersecretory lesions/tumour: This group of diseases needs to be distinguished from TCCRP because of the prominent follicular changes in the thyroid. One case of TCCRP reported by Colella et al. showed a cystic cavity containing glia-like secretion, papillary hyperplasia of columnar cells in the local cystic cavity, nuclear sulcus and intranuclear pseudoinclusion bodies under the microscope, but IHC showed that the tumour was mainly in situ, so the possibility of cystic hypersecretory carcinoma *in situ* with microinvasion could not be excluded (6). Detection of IDH2 gene mutations plays an important role in differential diagnosis.

TCCRP exhibits unique clinicopathological characteristics and is a low-invasive tumour. Most patients experienced no tumour recurrence during the follow-up period. One patient in the literature had internal mammary lymph node metastasis but survived without tumour recurrence for 10 years. Another patient had local recurrence and axillary lymph node metastasis after 5 years of follow-up, but survived without tumor for 4 years after the second operation (9). Cameselle et al. reported a case of a stage IIIc (pT2N3bM0) tumour recurrence that developed bone metastasis after 32 months of follow-up (2). However, Bhargava et al. questioned that it might be a common ER-positive breast cancer (10). Due to its indolent biological behaviour and good prognosis, some scholars support no aggressive clinical management for TCCRP (9).

In summary, TCCRP is an uncommon invasive carcinoma subtype with indolent clinical behavior found in the breast. Its histological morphology resembles that of tall cell subtype papillary thyroid carcinoma. Accurate diagnosis requires the integration of histomorphological assessment along with immunohistochemistry and molecular genetics analysis.

## Data availability statement

The original contributions presented in the study are included in the article/supplementary material. Further inquiries can be directed to the corresponding author.

## Ethics statement

The studies involving humans were approved by The Ethics Committee of the First Affiliated Hospital of Kunming Medical University. The studies were conducted in accordance with the local legislation and institutional requirements. The participants provided their written informed consent to participate in this study. The requirement of ethical approval was waived by Ethics Committee of the First Affiliated Hospital of Kunming Medical University for the studies involving animals because Subject information was recorded by means of identifiers. The studies were conducted in accordance with the local legislation and institutional requirements. Written informed consent was obtained from the individual(s) for the publication of any potentially identifiable images or data included in this article.

## Author contributions

ZL: Writing – original draft. YW: Writing – review & editing. ZW: Data curation, Writing – review & editing. CY: Conceptualization, Methodology, Project administration, Writing – review & editing. GP: Funding acquisition, Supervision, Validation, Writing – review & editing.
